# WWOSC 2014: Research Needs for Better Health Resilience to Weather Hazards

**DOI:** 10.3390/ijerph120302895

**Published:** 2015-03-05

**Authors:** Michel Jancloes, Vidya Anderson, Pierre Gosselin, Carol Mee, Nicholas J. Chong

**Affiliations:** 1 Health and Climate Foundation, Washington, DC 20005, USA; 2 Ministry of Health & Long-Term Care, 393 University Avenue, Toronto, ON M7A 2S1, Canada; E-Mail: vidya.anderson@ontario.ca; 3 Institut National de Santé Publique du Québec et Ouranos, 945 rue Wolfe, QC G1V 5B3, Canada; E-Mail: pierre.gosselin@inspq.qc.ca; 4 Toronto Public Health, 277 Victoria Street, Toronto, ON M5B 1W2, Canada; E-Mail: cmee@toronto.ca; 5 Trinity College, 6 Hoskin Avenue, Toronto, ON M5S 1H8, Canada; E-Mail: nicholas.chong@mail.utoronto.ca

**Keywords:** public health, environmental health, weather hazards, risk vulnerability, health emergencies, extreme weather events, weather forecasting, adaptive response

## Abstract

The first World Weather Open Science Conference (WWOSC, held from 17–21 August 2014 in Montreal, Québec), provided an open forum where the experience and perspective of a variety of weather information providers and users was combined with the latest application advances in social sciences. A special session devoted to health focused on how best the most recent weather information and communication technologies (ICT) could improve the health emergency responses to disasters resulting from natural hazards. Speakers from a plenary presentation and its corresponding panel shared lessons learnt from different international multidisciplinary initiatives against weather-related epidemics, such as malaria, leptospirosis and meningitis and from public health responses to floods and heat waves such as in Ontario and Quebec, Canada. Participants could bear witness to recent progress made in the use of forecasting tools and in the application of increased spatiotemporal resolutions in the management of weather related health risks through anticipative interventions, early alert and warning and early responses especially by vulnerable groups. There was an agreement that resilience to weather hazards is best developed based on evidence of their health impact and when, at local level, there is a close interaction between health care providers, epidemiologists, climate services, public health authorities and communities. Using near real time health data (such as hospital admission, disease incidence monitoring…) combined with weather information has been recommended to appraise the relevance of decisions and the effectiveness of interventions and to make adjustments when needed. It also helps appraising how people may be more or less vulnerable to a particular hazard depending on the resilience infrastructures and services. This session was mainly attended by climate, environment and social scientists from North American and European countries. Producing a commentary appears to be an effective way to share this session’s conclusions to research institutions and public health experts worldwide. It also advocates for better linking operational research and decision making and for appraising the impact of ICT and public health interventions on health.

## 1. Background

The Intergovernmental Panel on Climate Change, in its most recent review and synthesis of available research, provided ample and compelling evidence that the severity, length and frequency of various types of extreme weather events will increase in the coming half- century [1]. In preparation and response to the urgency demanded by the predictions, many health experts have examined the intricate yet clear link between climate events and their health implications [2]. In efforts to assemble a comprehensive adaptive strategy, there is a need for dialogue and interaction between health and climate services.

For years, cross-disciplinary research and dialogue between meteorologists, climate modelers and health scientists has been the object of important efforts at international and national levels. The Regional Integrated Science and Assessment programme of the National Oceanic and Atmospheric Administration in the United States is a good example [3]. The development of high quality climate services with new information technologies has helped forecasting climate sensitive health risks, having in mind more adequate planning and budget allocation for health [4].

Moving from risk assessment towards risk management, the dialogue has been transformed into interaction for supporting public health decision, especially for implementing local interventions in countries, like Bhutan, Brazil, Cambodia, Canada, China, Ethiopia, Indonesia, Madagascar, Nicaragua, Niger, the United Kingdom, and the U.S.A.

Weather Information and Communication services have been more and more linked with epidemiological surveillance, vulnerability assessment, access to health care, peoples’ behaviour, one health approach, prevention interventions…Their application has covered a large spectrum of health risks related to a particular hazard, such as risks associated to socio-economic conditions, including access to water, food, shelter conditions, spread of diseases and access to health infrastructure and services.

Sharing experiences, developing capacities and institution building, have increasingly been on the agenda of governments. International cooperation programmes, include for example the “Climate Information for Public Health Action Network” (Columbia University, USA). A multitude of new alliances- such as “The Global Climate and Health Alliance”, service partnerships, like the “Climate Services Partnership”, or institution networks and foundations has emerged to develop and promote the use of these weather/climate ICT. Tremendous advances in ICT inform now health risk management, not only to prepare forecasting tools but also to improve the validity and spatiotemporal applicability of weather information. Linking risk exposure and vulnerability mapping, provides the potential for better informed decision making and for local-level implementation [5].

There is a momentum in the development of adaptive strategies which support the argument that the arena for a worldwide conference is needed and could have significant outcomes. Within this frame, the first World Weather Open Science Conference (WWOSC), held in Montreal from 17 to 21 August 2014 [6], was one of the largest international multidisciplinary meetings of meteorologists and user communities. The agenda included user community and social scientists perspectives and different panel sessions covering energy, insurance, multidisciplinary research, sociological aspects of disaster management and health.

## 2. Climate and Health at the WWOSC

The plenary session on “Research Needs for Better Health Resilience to Weather Hazards” presented few experiences from the Health and Climate Foundation and on global multidisciplinary networks that have addressed weather-related epidemics (the Global Leptospirosis Environmental Action Network, GLEAN, and the Meningitis Environmental Risk Information Technologies, MERIT)

The following panel introduced a Canada case story with three complementary contributions on experiences from Toronto at municipal level and from Ontario and Quebec at provincial level. Vidya Anderson addressed responses to weather events at provincial-level, particularly discussing the framework for action of the Ontario government to events of flooding, ice storms, and heat stress. The action plan was implemented at policy-level through legislative mandates, ensuring the high accessibility and quality of information. Pierre Gosselin, panellist, examined the specific tools used by the Quebec government for strengthening the surveillance services, the SUPREME system, and for to developing experience-based responses for future events [7]. The SUPREME system consists of incorporating ICTs as an open source, real-time, web service to access data from several sources. Highlighting the success of the project implementation, Gosselin identified the importance of strengthening communication between weather scientists and public health service officials. Carol Mee explored the issue of public health response by identifying how information is accessed and interpreted by the user population; the locally-embedded approach requires an understanding of the knowledge translation process.

It was recognized, however, that this case of a highly developed city with a highly committed population, may not be appropriate to address climate issues in different cultural, social and environmental contexts. Sharing experiences and lessons learnt as well as developing capacities, including research and institution building needs be on the agenda of bilateral and international cooperation arrangements. The current implementation and use of a simplified SUPREME system in Niamey (Niger) and Morocco is an example [8].

Contributions from panel experts and participants (mainly climate and environment experts, social scientists and few public health and policy makers) were very much action oriented. They mainly focused on the best use of ICTs to inform decision making processes.

## 3. Discussions

Over the last decade, the rapid development of climate services and meteorological offices was witnessed in quantity and quality. These services are processing and analyzing a huge collection of weather information in order to develop a database accessible and manageable for knowledge management at community level. The data, if properly developed as a user-interactive database, have the potential to provide information for both pre-emptive preparation and post-event response. The challenge is then, to tailor the climate services so as they become more relevant and useful in specific health related decision- making. It was observed in an increased number of countries, that climate services and health surveillance offices, with the support from research institutions, interact by combining near real time health data (such hospital admission, disease incidence monitoring…) and uptake of climate/weather information. This is particularly relevant for planning for and responding to extreme weather events. In this regard:

- ICT information needs be provided to communities engaged in anticipative intervention;
- Targeted ICT must involve essential actors and stakeholders in climate-health issues in order to ensure the inclusion of marginalized or vulnerable populations.

This interaction requires strengthening their respective managerial and institutional capacities in line with their available resources. It consists of an incremental process based on steady feedback mechanisms. This process also includes appraising the relevance of decision, the usefulness and predictive value and of weather information as well as the effectiveness of interventions. It also helps identifying the vulnerability of a population to a particular hazard depending on the resilience infrastructure and services.

Related to the implementation aspects, the use of ICTs is a challenge for the public health practitioners and includes:

- Strengthening the quality of epidemiological surveillance and of real time health data on vulnerable population groups exposed to a particular hazard.
- Improving the synergy between observational experiments and modelling for policy guidance

Enhancing community resilience to weather hazards through a social process should be developed by:

- Community participation based on knowledge mobilization and on evidence-based approach rooted in health science and epidemiology strengthen the preparatory responses to oncoming weather events.
- Resilience to weather hazards is best developed with evidence of health impact when, at local level, climate services, epidemiologists, health care providers, public health authorities and communities interact.

The WWOSC participants also pointed out the need for:

- Time and space downscaling of accurate weather information for its applicability in public health decision making at local level
- Validation of models and new public information and communication technologies to improve the preparedness response, early warning and alert management and to promote anticipative intervention awareness

## 4. Conclusions

This WWOSC session was designed to put the scene and advocate for interdisciplinary dialogue and interaction between experts with climate and weather background, health professionals and community users. The same is progressively happening at country level and has to grow through structured interface of expertise and responsibilities.

This “Commentary” is mainly addressed to climate services, public health officials and multidisciplinary research networks over the world. It gives the main messages from this significant conference and encourages them to develop an interaction frame which fits their own social, environmental and operational context. The main objective is to build better health resilience to weather hazards. It calls for a research agenda which could make this interaction frame most effective by taking advantage of weather ICT advances and of health resilience building experiences. The continuation of dialogue between the medical and public health disciplines with weather/climate specialists, should lead to future research activities focusing on the following critical tracks. Specific recommendations were issued to further enhance operational research on health resilience to weather hazards; there is need to:

(1) Address the proper translation of weather/climate technology data for spatiotemporal applicability at public health decision making levels for local implementation needs;
(2) Produce adequate information and communication with community users for better health resilience and vulnerability reduction to a particular weather hazard;
(3) Combine near real time health and weather data to get feedback on effectiveness of prevention and response interventions, including vulnerability shifts;
(4) Stimulate dialogue between decision makers and key actors to integrate climate technologies and new information /communication tools (such as GIS and remote sensing) into the health sector policies and plans, in a manner that is self-evaluating, self-growing, and accessible.

It is expected that the next WWOSC could provide scientific evidence of progress on health resilience by communities against different types of weather hazard and put health at the centre of adaptation strategies.
